# Capsaicin and TRPV1: A Novel Therapeutic Approach to Mitigate Vascular Aging

**DOI:** 10.14336/AD.2024.1292

**Published:** 2026-02-08

**Authors:** Xing-Yu Cui, Jun-Kun Zhan

**Affiliations:** ^1^Department of Geriatrics, The Second Xiangya Hospital, Central South University, Changsha, Hunan, China; ^2^Institute of Aging and Age-related Disease Research, Central South University, Changsha, Hunan, China

**Keywords:** Capsaicin, TRPV1, Vascular aging, Therapeutic agent

## Abstract

Vascular aging and its associated diseases represent a principal cause of mortality among the global elderly population, making the mitigation of vascular aging a significant aspiration for humanity. This article explores the intersection of nature and health, focusing on the role of the natural plant, pepper, and its principal bioactive compound, capsaicin, in combating vascular aging. By examining molecular and cellular mechanisms as well as phenotypic alterations in blood vessels, we offer a comprehensive review of the effects of capsaicin and its receptor, transient receptor potential vanilloid 1 (TRPV1), within vascular aging. We propose that capsaicin may serve as the medication with the potential to slow the progress of vascular aging and could constitute a new strategy to treat vascular aging related disease.

## 1. Introduction

### 1.1 Pathophysiology of Vascular Aging

**Figure 1.**
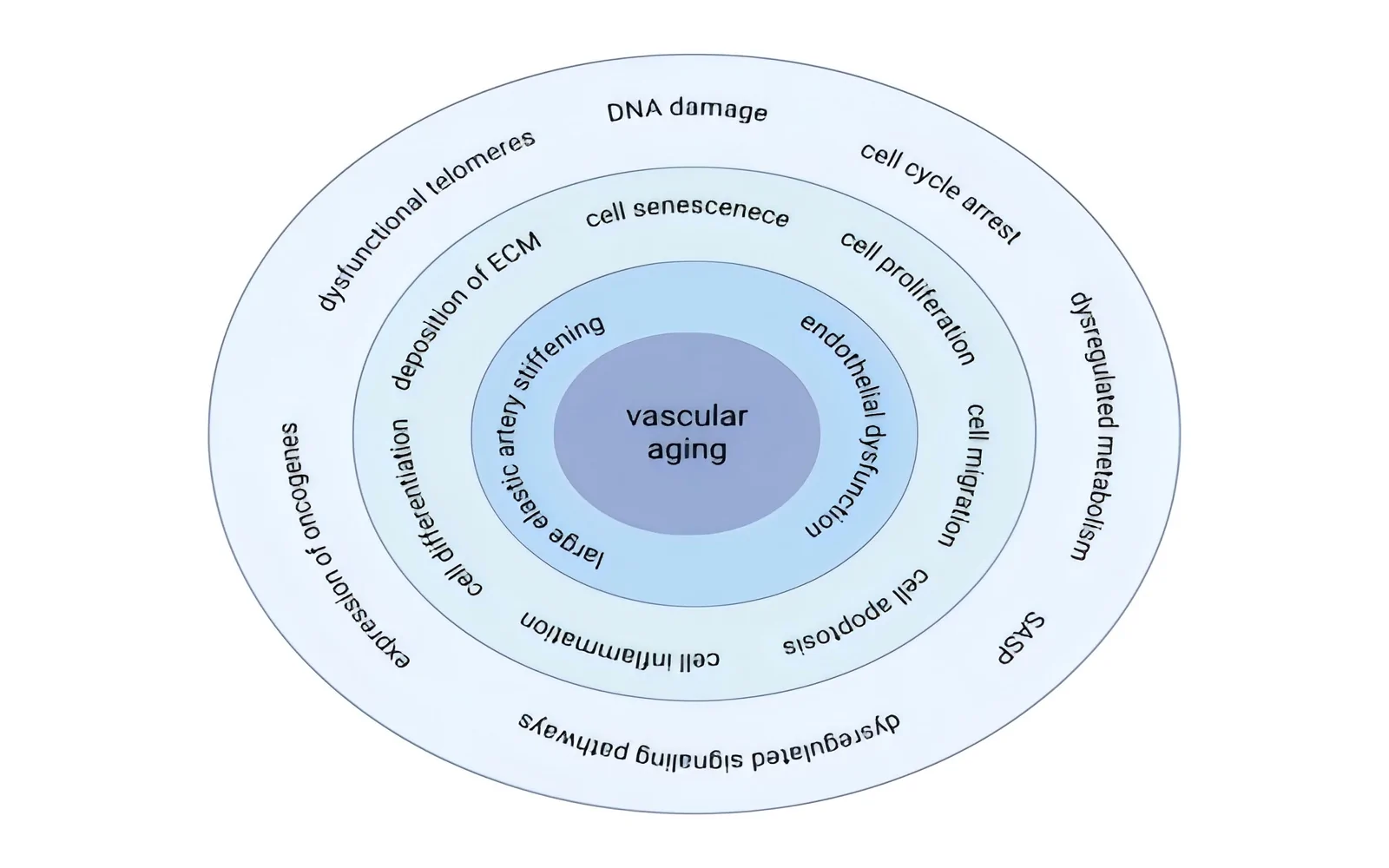
**The mechanisms of vascular aging**. Vascular aging is influenced by various stress factors and dysregulated signaling pathways, including dysfunction of telomeres, DNA damage, expression of specific oncogenes, cell cycle arrest, disrupted metabolism, and SASP. These factors promote cell senescence, which is characterized by transitions involving proliferation, migration, apoptosis, inflammation, and differentiation. Collectively, these changes contribute to endothelial dysfunction and the stiffening of large elastic arteries, ultimately leading to vascular aging.

A population-based study conducted in the US indicates that age represents the most significant cardiovascular risk factor, overshadowing the effects of traditional risk factors [1]. Aging can lead to vascular aging, a common pathological mechanism underlying the progression of diverse cardiovascular and cerebrovascular disorders. Studies have demonstrated that reversing the aging phenotype of blood vessels, such as improving capillary density, can reverse organ senescence and effectively reduce the incidence of aging-related diseases [2]. To intervene in aging, a comprehensive understanding of vascular aging is indispensable (Fig 1).

Endothelial dysfunction and large elastic artery stiffening are the two major age-related arterial phenotypes [3]. To assess their significance, it is necessary to understand the structure of the vascular system. Blood vessels are a series of tubes through which blood flows, with the walls generally comprising three-layer structures, namely the intima, media, and adventitia, arranged sequentially from the luminal side outwards. Endothelial cells (ECs), located in the inside of the vessel wall, and vascular smooth muscle cells (VSMCs), situated on the outside of ECs, represent the phenotypic changes associated with vascular aging. ECs are positioned between plasma and vascular tissues, playing pivotal roles in facilitating the delivery of oxygen and nutrients, maintaining blood fluidity, regulating vascular permeability, and maintaining tissue homeostasis [4]. VSMCs are primarily responsible for contracting and regulating the tone and diameter of blood vessels, as well as regulating blood pressure and flow [5]. Adventitia primarily consists of elastic and collagen fibers; however, it has been largely overlooked. The adventitial stroma is the most complex segment of the vascular structure, which comprises an extracellular matrix (ECM) framework that includes fibroblasts, blood vessels, lymphatic vessels, nerves and so on [6]. Typically, it participates in regulating vascular tone and nutrient supply. However, recent research has demonstrated that the adventitia plays a pivatol part in immune responses, orchestrating chronic inflammation [7], and functioning as a storage for progenitor cells, which are critically functional in vascular repair after various pathological stimuli [8]. In summary, the proliferation and migration of VSMCs, along with impaired integrity of elastin fibers and ECM deposition, contribute to the stiffening of large elastic arteries and exacerbate endothelial dysfunction [9].

Recent studies specify that cellular senescence is a common molecular and cellular mechanism for age-related macrovascular and microvascular lesions and diseases [10], especially EC senescence [11]. Cellular senescence, observed in the cells of the aged, also in cells stimulated by various stress factors, including the dysfunction of telomeres, DNA damage, dysregulation of miRNAs, and expression of certain oncogenes [12-14]. Diverse cell types and numerous signaling pathways participate in the progress of cell senescence [15]. These include cell cycle arrest, dysregulated metabolism, and the senescence-associated secretory phenotype (SASP) [11, 15]. Other common signaling pathways include those related to growth factors, klotho, and nutrient sensing pathways such as mammalian Target of Rapamycin (mTOR), adenosine monophosphate protein kinase (AMPK), and sirtuin-1 (SIRT1) [3]. Senescent cells elicit a sophistcated pro-inflammatory response termed as SASP, which comprises a series of pro-inflammatory cytokines [16]. In this way, senescent cells can influence the functionality and phenotypic characteristics of adjacent cells within the vascular system through paracrine and autocrine secretion, undergoing transitions encompassing proliferation, migration, apoptosis, inflammation, and differentiation, leading to cellular dysfunction and ECM remodeling, thereby contributing to the progression of both macro- and microvascular diseases related with aging [17].

### 1.2 Capsaicin, TRPV1 and Vascular Aging

**Figure 2.**
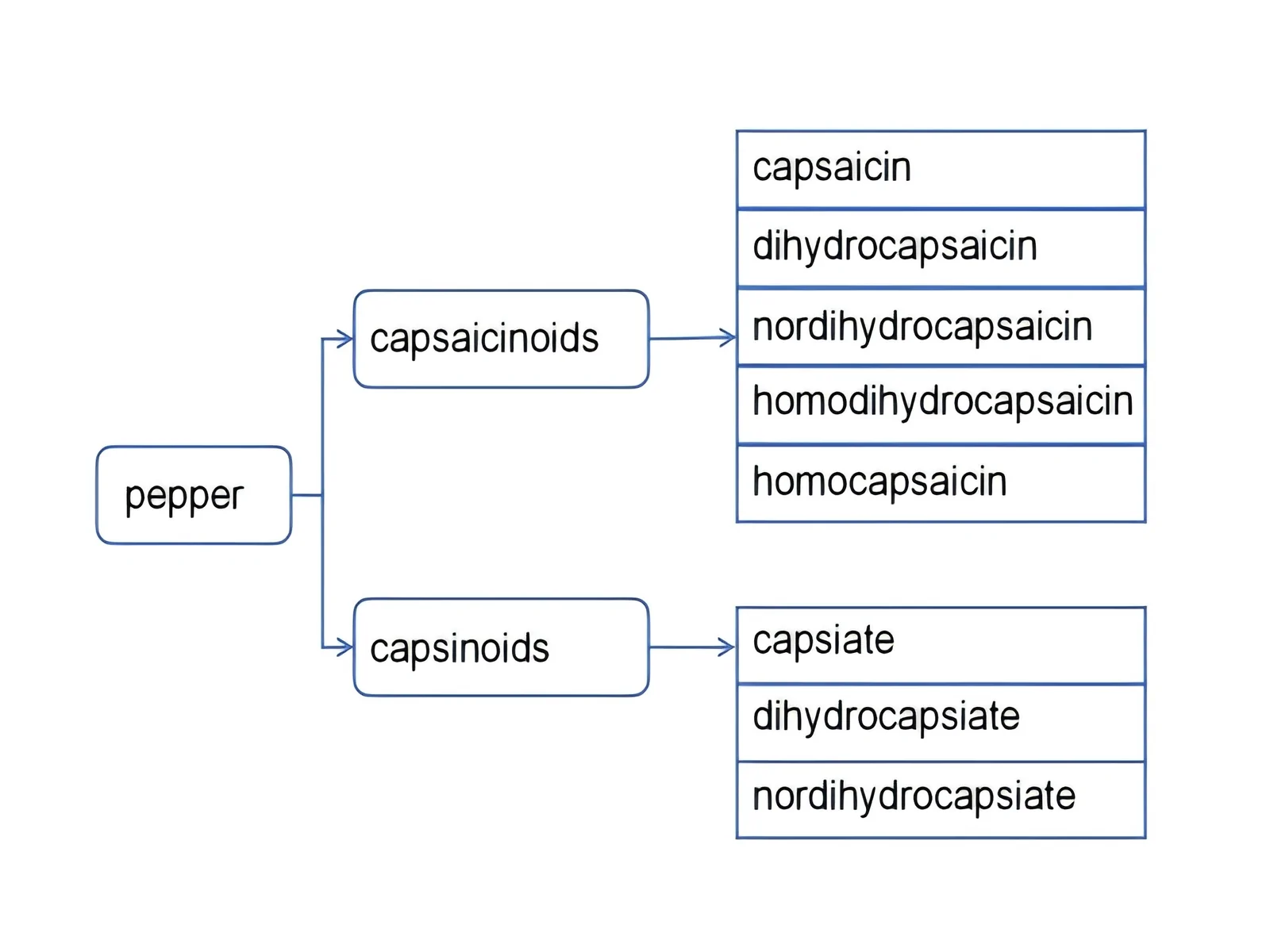
**Diverse array of compounds in peppers**. Capsaicinoids and capsinoids are the two major categories in peppers. Among them, Capsaicinoids encompass capsaicin, dihydrocapsaicin, nordihydrocapsaicin, homodihydrocapsaicin, and homocapsaicin; whereas capsinoids consist of capsiate, dihydrocapsiate, and nordihydrocapsiate.

Globally, spicy cuisines, particularly those featuring fiery chili peppers, are consumed in significant quantities. Upon consumption of red peppers, individuals experience a pronounced sensation of heat, primarily due to the main pungent alkaloid present in these peppers, capsaicin. Capsaicin is synthesized in chili peppers through the incorporation of a branched-chain fatty acid into vanillylamine, with the molecular formula C_18_H_27_NO_3_ [18]. Capsaicin is part of a broader class known as capsaicinoids, which includes various compounds beyond capsaicin itself, such as dihydrocapsaicin, nordihydro-capsaicin, homodihydrocapsaicin, and homocapsaicin, each contributing to the unique pungency profile of peppers [19]. Additionally, peppers contain non-pungent analogues of capsaicin, collectively termed capsinoids, which include capsiate, dihydrocapsiate, and nordihydrocapsiate [20] (Fig 2). Capsinoids are esters of vanillyl alcohol and fatty acids, with the molecular formula C_18_H_26_O_4_ [21]. Although capsaicinoids and capsinoids exhibit similar structural characteristics, they differ in their central linkages, enhancing our understanding of their chemical relationships.

Capsaicin serves as the primary spicy component in chili peppers and exhibits various biological activities. Notably, capsaicin holds a pivotal role in addressing metabolic disorders. The dysregulation of glucose homeostasis is intimately associated with numerous metabolic syndromes, including insulin insensitivity, obesity, type 2 diabetes mellitus, and cardiovascular diseases. Research indicates that regular consumption of chili peppers containing capsaicin effectively ameliorates postprandial hyperglycemia and hyperinsulinemia, as well as fasting lipid metabolic abnormalities [22]. Furthermore, in Chinese adults, chili pepper intake is negatively correlated with the ratio of overweight and obesity and demonstrates beneficial effects in reducing hypertension and cardiac hypertrophy [23, 24]. Moreover, capsaicin possesses anti-cancer, anti-inflammatory, and antioxidant properties and is used as a topical analgesic [25]. Metabolic imbalance is a significant risk factor for vascular senescence. Although specific research on capsaicin's role in this aging process remains limited, there is a strong belief in its potential benefits for mitigating vascular aging.

**Figure 3.**
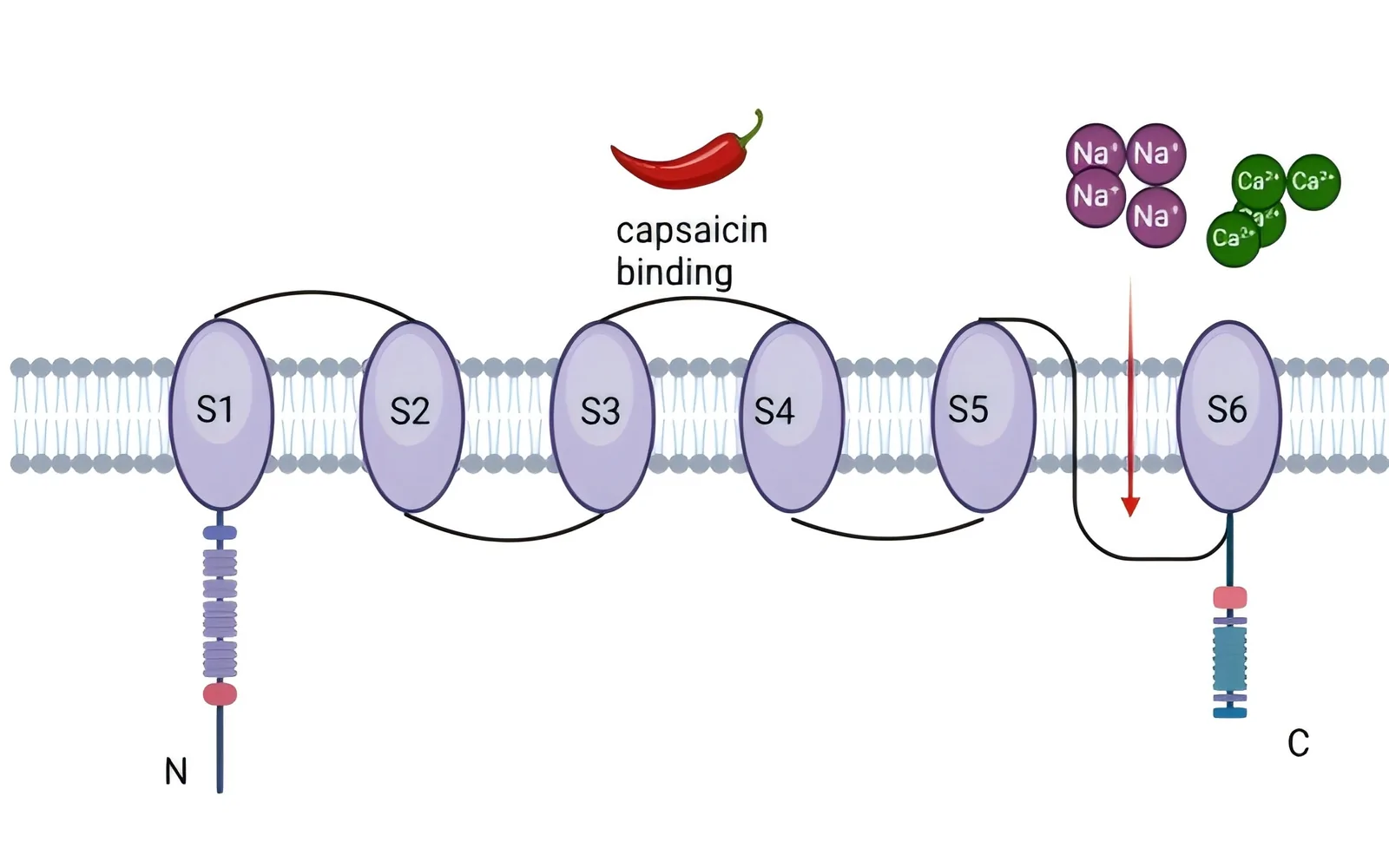
**Capsaicin and TRPV1**. TRPV1, a protein comprising six transmembrane domains (S1-S6), with both N and C termini of each subunit located intracellularly. Capsaicin engages with S3 and S4, activating TRPV1, sodium and calcium ions flowing through the intervening loop situated between S5 and S6 into the cell, leading to the sensation of spiciness and pain.

For many years, scientists have been continuously studying the mechanisms of action of capsaicin, culminating in the identification of major capsaicin-sensing genes and the ion channel proteins they encode. The Transient Receptor Potential Vanilloid type 1 (TRPV1) channel protein, can be activated by a spectrum of agonists, inclusive of acid (pH < 6.5), heat, and capsinoids [26]. Studies demonstrate that capsaicin directly activates TRPV1 by binding to intracellular sites within the channel protein [27, 28], subsequently activating numerous physiological pathways. TRPV1, a non-selective cation channel, preferentially conducts Ca2+ ions, with both the N- and C-termini of each subunit positioned intracellularly [29]. It is structured as a protein comprising six transmembrane domains (S1-S6), interconnected by intracellular and extracellular loops, with the crucial pore or ion-conducting pathway formed by the loop situated between S5 and S6 [30]. Outside the cell, capsaicin interacts with the S3 and S4. Upon binding, capsaicin adopts a "tail-upwards, head-downwards" orientation, firmly tether the ligand to the receptor [26]. Upon activation of TRPV1 by capsaicin, sodium and calcium ions permeate into the cell through TRPV1 channel, resulting depolarization of nociceptive neurons, action potential firing, and final sensations of spiciness and pain [29] (Fig 3).

TRPV1 was first discovered in primary afferent nociceptive neurons located in the dorsal root ganglia (DRGs), trigeminal ganglia (TG), and vagal ganglia [31]. Recently, studies have revealed that various non-neuronal cells, including endothelial cells [31], arteriolar SMCs [32], and adipose tissue [33], also express TRPV1. It has been shown that activating TRPV1can reduce vascular lipid accumulation and attenuate atherosclerosis [34]. As the potential therapeutic targets for cardiovascular diseases, TRPV1 is receiving increasing attention [35]. In 2010, a study reported that dietary capsaicin can chronically activate TRPV1, augment the phosphorylation of protein kinase A (PKA) and endothelial NO synthase (eNOS), thereby enhancing the production of NO production in ECs, facilitating vasorelaxation, and reducing blood pressure [36]. Furthermore, studies indicate that capsaicin suppresses ECs senescence [37], inhibits osteogenic transdifferentiation of VSMCs, and prevent arterial calcification [38] via TRPV1. Besides capsaicin, citropten can also inhibit the proliferation and migration of VSMCs by activating TRPV1 [39]. Thus, capsaicin may significantly alleviate vascular aging through the TRPV1 channel.

However, TRPV1 receptor is not the sole target of capsaicin. A study indicates that topical application of capsaicin induces increased blood flow by releasing vasodilator neuropeptides, but capsaicin-induced relaxation was unaffected by TRPV1 inhibition [40].This suggests that capsaicin may exert its effects through other non-TRPV1 pathways. Recent studies have summarized that capsaicin can exert direct modulation over the functions of voltage-gated Na+, K+, and Ca2+ channels, as well as ligand-gated ion channels and an array of other ion transporters and enzymes implicated in cellular excitability [41, 42]. However, most of these studies have been conducted in non-vascular cells, such as cardiomyocytes, neuronal cells, and islet cells. Regarding vascular effects, the application of capsaicin (1 mM) has been shown to exert a relaxing effect in rat aortic rings, which is related to the inhibition of the voltage-dependent L-type Ca2+ channel [43] and voltage-dependent K+ channel [44]. Thus, the TRPV1-independent effects of capsaicin also deserve more attention.

Additionally, we observed a particular substance, calcitonin gene-related peptide (CGRP), which is recognized as a pivotal factor in the initiation and exacerbation of migraines and trigeminal-autonomic headaches [45]. Upon binding to TRPV1, capsaicin induces the release of CGRP from sensory nerve terminals [46]. CGRP correlates profoundly with vascular functionality, exhibiting vasodilatory effects, particularly in cerebral circulation [47-49]. CGRP also promotes neovascularization in various significant pathological conditions and signals the vascular system [50]. In experiments, capsaicin functions as a sensory nerve activator that releases perivascular CGRP to induce vasodilation [51]. Therefore, we posit that capsaicin can exert its vasodilatory effects through CGRP, and investigating the relationship between CGRP and vascular aging may yield valuable insights.

Capsiate is the principal component of capsinoids. However, its biological activities are relatively understudied compared to those of capsaicin. A comprehensive meta-analysis of human trials demonstrates that consumption of capsinoids results in heightened energy expenditure and increased fat oxidation, particularly in individuals with a BMI exceeding 25 kg/m² [52]. Simultaneously, research confirms that capsiate suppresses adipogenesis and improves insulin sensitivity both in vitro and in vivo [53, 54]. Furthermore, the gut microbiota metabolite capsiate inhibits ferroptosis through TRPV1 activation in the context of intestinal ischemia/reperfusion injury, presenting a promising therapeutic strategy for managing this condition [55]. Recent investigations of capsiate primarily focus on the role in addressing obesity, metabolic disorders, carcinomas, and gastrointestinal ailments [56]. In the context of vascular aging, prior research demonstrates the efficacy of capsiate in hindering pathological angiogenesis and mitigating vascular permeability [57], underscoring its positive role in vascular health and aging. Given the beneficial impact of capsiate on metabolic processes, its significance in the context of vascular aging merits further exploration.

## 2. The Role of Capsaicin in Aged Arterial Phenotypes

Increased stiffness of large elastic arteries and endothelial dysfunction constitute two arterial phenotypes intimately linked to aging. These phenomena serve as significant precursors for diagnosing future cardiovascular diseases (CVDs) and are predictors of CVD-associated morbidity and mortality [58]. Accordingly, this article will begin by exploring aged arterial phenotypes and elucidating the macroscopic effects of capsaicin on vascular aging.

### 2.1 Capsaicin and Endothelial Dysfunction

Endothelial dysfunction is pivotal in most vascular pathologies and vascular aging [59]. Studies show that capsaicin can reduce endothelial dysfunction [60, 61]. ECs maintain vascular tone, modulate oxidative stress, and regulate the local activity of angiotensin II through the release of NO, prostacyclin (PGI2), and endothelin. NO and PGI2 are potent vasodilators, while eNOS is responsible for their normal production [62]. Evidence suggests that dietary capsaicin increases the production of NO and endothelial NO synthase in ECs [36, 63]. TRPV1 activation in ECs also increases NO production [64] and activates eNOS [65], contributing to endothelial relaxation and attenuating inflammatory responses [66]. Furthermore, capsaicin exerts its vasodilatory effects on mesenteric arterioles by eliciting the production of PGI2 [67]. Beyond these aspects, ECs play a crucial role in maintaining vascular barrier function and actively participating in angiogenesis. Impairment of endothelial function can lead to increased vascular permeability and enhanced angiogenesis. Capsaicin has been demonstrated to suppress the enhanced paracellular permeability induced by high glucose [68]. It also exerts inhibitory effects on angiogenesis by suppressing vascular endothelial growth factor (VEGF)-mediated proliferation, DNA synthesis, chemotactic migration, and capillary-like tube morphogenesis in primary cultures of human ECs [69]. In summary, capsaicin is beneficial in improving endothelial dysfunction.

### 2.3 Capsaicin and large Elastic Artery Stiffening

Large artery stiffening is believed to impact CVDs in several ways, leading to higher systolic pressure, reduced diastolic pressure, and widening of pulse pressure [70], changing blood flow distribution, particularly in brain and kidney [71, 72]. The mechanisms leading to large elastic artery stiffening include VSMC proliferation and migration, compromised integrity of elastin fibers, and deposition of ECM [9]. Research indicates that chronic hypoxia markedly augments the proliferative capacity of PASMCs via the TRPV1 channel [73], and activation of TRPV1 has been corroborated to elicit the migration of PASMCs [74]. However, researchers remain hesitant to acknowledge TRPV1 function in promoting the proliferative migration of VSMCs within aortic vessels due to the heterogeneous nature of VSMCs across different tissue types. They speculate that TRPV1 activation may instead inhibit the proliferative migration of VSMCs [75, 76]. Both Capsaicin and activation of TRPV1can facilitate CGRP secretion [77-79]. Peripheral nerves release CGRP, which inhibits the proliferation of aortic VSMCs, achieved through an increase in cyclic adenosine monophosphate (cAMP) levels [80]. Additionally, endogenous CGRP can inhibit oxidative stress and the proliferation of VSMC induced by vascular injury [81]. Moreover, capsaicin and TRPV1 can activate the AMPK pathway [82, 83]. AMPK has been shown to exert inhibitory effects on the proliferation and migration of VSMC, and vascular remodeling following injury [84]. Therefore, the theoretical function of suppressing VSMC proliferation and migration through capsaicin-mediated TRPV1 activation is plausible but requires further empirical validation through rigorous experimentation. Studies on the impaired integrity of elastin fibers and ECM deposition appear to be relatively scarce. Suppressing TRPV1 amplifies ECM protein expression, while capsaicin can partially mitigate this effect in hepatic stellate cells [85]. Additionally, capsaicin induces reduced ECM deposition in hepatic stellate cells [86]. In colonic tissue, capsaicin exposure increases collagen and elastin fiber accumulation, intensifies ECM remodeling, and exacerbates tissue stiffness [87]. However, TRPV1 activation mitigates ECM protein production and slows the progression of renal fibrosis [88]. In summary, evidence suggests that capsaicin can reduce ECM deposition and elastin fiber accumulation; however, further research on ECM in vascular tissues is essential for conclusive evidence.

## 3. The Role of Capsaicin in the Cellular Mechanism of Vascular Aging

Cell senescence is a hallmark of aging, with shared cellular mechanisms underlying macrovascular and microvascular diseases [10]. Aged cells undergo the SASP and transform into various cellular phenotypes, including proliferation, migration, apoptosis, and inflammation, ultimately manifesting as an aged arterial phenotype [10]. Next, we explore the cellular mechanisms to elucidate the effects of capsaicin on vascular aging (Table 1).

**Table 1:** The role of capsaicin in cellular mechanism of vascular aging.

Cellular phenotypes	Effect	Mechanism/Condition
**cell senescence**	inhibit	by elevating SIRT1 levels [37] by suppressing NADPH oxidase and reducing reactive oxygen species [89] by retarding mitochondrial dysfunction [91]
**cell proliferation and migration**	inhibit	in the VEGF-induced primary cultured human ECs [69]
	promote	in mice with wire-mediated injury to the carotid artery [92] in rat hind limb ischemic model [93, 94]
**cell apoptosis**	inhibit	by mitigating oxidative and nitrative stress [98] by inhibiting MAPK pathways [100] by inhibiting ERK1/2-NOX4 [101]
**cell inflammation**	inhibit	by activating eNOS [66]
**cell calcification**	inhibit	by inhibiting the Wnt/β-catenin signaling pathway [107] by upregulating SIRT6 [38]

### 3.1 Capsaicin and Cell Senescence

EC senescence is a significant contributor to vascular aging and associated diseases [11]. Capsaicin suppresses high glucose-mediated endothelial cell senescence by elevating SIRT1 levels [37]. Additionally, CGRP exerts an antagonistic effect on angiotensin II (Ang II)-mediated endothelial progenitor cell (EPC) senescence by suppressing NADPH oxidase expression and reducing reactive oxygen species generation [89]. CGRP remains effective against EPC senescence in hypertension [90]. Another, capsaicin retards mitochondrial dysfunction, thereby impeding cellular aging processes, by modulating the mitochondrial membrane protein SLC25A12 in VSMCs [91]. Therefore, the use of capsaicin can effectively delay cellular senescence.

### 3.2 Capsaicin and Cell Proliferation and Migration

When vascular injury occurs, the proliferation and migration of ECs promote re-endothelialization, facilitating the restoration of damaged vasculature. Conversely, VSMCs and ECs proliferation and migration contribute to intimal thickening, ultimately leading to vascular aging and stenosis. Importantly, the effects of capsaicin are not constant. Following wire-mediated injury to the carotid artery in mice, capsaicin significantly promotes ECs’proliferation and migration, conversely, does not involve VSMCs, hence promote the heal of injury [92]. In an ischemia model, TRPV1 activation through capsaicin treatment enhances aortic EC proliferation, ultimately contributing to collateral vessel growth [93, 94]. However, in rat aortic ring assay, capsaicin inhibits proliferation of ECs, demonstrate its antiangiogenic activity [69]. Capsaicin’s inhibitory effects on the proliferation and migration of VSMC have been previously summarized in the former text. With its effects on ECs, this suggests that capsaicin can help modulate the vascular system toward a normal physiological state by regulating cellular proliferation and migration, across different vascular states.

### 3.3 Capsaicin and Cell Apoptosis

In tumor diseases, capsaicin is recognized to induce apoptosis in carcinoma cells [95, 96]. Consequently, it has been frequently studied as a potential cancer treatment by inhibiting cell apoptosis. However, researchers have suggested that utilizing natural medicines to suppress cell apoptosis may represent a promising approach for treating vascular diseases [97]. In cardiac microvascular ECs, the activation of TRPV1 by capsaicin protects against diabetes-induced EC apoptosis by alleviating oxidative and nitrative stress [98]. In contrast to ECs treated with oxidized low-density lipoprotein (ox-LDL), apoptotic protein expression in ECs treated with capsaicin is significantly reduced [99]. These studies provide direct evidence for the anti-apoptotic properties of capsaicin. Furthermore, research indicates that CGRP plays anti-apoptotic effects on EPCs, possibly by inhibiting MAPK pathways [100]. Additional evidence shows that CGRP can mitigate high-glucose-induced cell apoptosis through inhibiting ERK1/2-NOX4 [101]. However, there is a scarcity of investigations exploring the effects of capsaicin on the apoptosis of VSMCs, primarily due to the prevailing notion that endothelial cell apoptosis is an initial event in various vascular-related diseases. Based on the limited research findings available, we propose that capsaicin may exhibit a degree of resistance to vascular cell apoptosis.

### 3.4 Capsaicin and Cell Inflammation

Inflammation is significant in the progression and perpetuation of cardiovascular disease [102]. Capsaicin has demonstrated the ability to prevent vascular disease by enhancing NO production and mitigating inflammatory responses [103]. Additionally, TRPV1 activation inhibits LPS-induced inflammatory response in ECs through the activation of eNOS, which is accompanied by a reduction in pro-inflammatory cytokine and chemokine production, reduced expression of adhesion molecules, and diminished monocyte adhesion [66]. Conversely, additional research has reiterated these findings from a contrasting perspective. In TRPV1-/- mice, inflammatory factors, such as IL-6, TNF-α, MCP-1, and MIP-2 are elevated [104]. It has been documented that oxLDL can induce the transformation of VSMCs to foam cells and upregulate pro-inflammatory molecules [105]. Coincidentally, TRPV1 activation has been shown to exert a mitigating effect on VSMCs treated with oxLDL [106]. Therefore, capsaicin may be beneficial to decrease vascular cell-associated inflammation.

### 3.5 Capsaicin and Cell Calcification

VSMCs calcification is a critical event for driving vascular calcification. Recent reports suggest that capsaicin inhibit the Wnt/β-catenin signaling pathway by upregulating TRPV1 receptor expression. This modulation leads to a reduction in the expression of Runx2 and BMP-2, ultimately contributing to the attenuation of VSMC calcification, as observed in mice [107]. Furthermore, capsaicin enhances the deacetylation and degradation of Hif1α through the upregulation of SIRT6, which reduces calcium deposition in VSMCs, thereby suppressing osteogenic transdifferentiation and mitigating the risk of arterial calcification [38]. Given the limited research available, we have identified the potential of capsaicin in inhibiting VSMCs calcification.

## 4. The Role of Capsaicin in the Molecular Mechanism of Vascular Aging

DNA damage, encompassing oxidative damage, strand breaks, DNA adducts, and telomere shortening [108], along with the ensuing diverse DNA damage response (DDR) pathways, significantly contributes to the process of vascular aging [109]. Current research predominantly focuses on the effects of capsaicin on tumors through the lens of DNA damage. This perspective posits that capsaicin may exert potential carcinogenicity by inducing oxidative DNA damage [110] and strand breaks [111]. However, capsaicin does not induce significant DNA strand breaks at concentrations ≥200 µM; conversely, at 175 µM, protein synthesis decreases 50% after 24 hours of incubation [112]. Given the heterogeneity of cells, the precise effect of capsaicin on DNA damage in vascular cells remains unclear and necessitates further experimentation specifically within ECs for validation. Mitochondria, serving as the cellular powerhouses, exhibit dysfunction as a hallmark of cell senescence, leading to the decline in organ, tissue, and overall body function [113]. Studies demonstrate that TRPV1 activation can upregulate UCP2, thereby ameliorating coronary dysfunction of atherosclerotic mice by alleviating endothelial mitochondrial dysfunction [114]. Notably, UCP2 is an adaptive antioxidant protein, preventing endothelial dysfunction triggered by mitochondrial ROS [115]. Numerous experiments have conclusively shown that TRPV1 is capable of upregulating UCP2 expression [116-118]. Concurrently, capsaicin has been shown to inhibit ROS and subsequently decline mitochondrial membrane potential, thus mitigating mitochondrial dysfunction [99]. On the other side, cell senescence is a condition of irreversible cell cycle arrest. The activation of cell cycle arrest due to oxidative stress can precipitate premature senescence in vascular cells [119]. Indeed, research has demonstrated that capsaicin facilitates the upregulation of SIRT1 expression while concurrently diminishing the expression of the senescence biomarker p21, thereby protecting ECs against premature senescence induced by insulin hyperglycemia (IHG) through the alleviation cell cycle arrest [37]. While there is a scarcity of articles exploring the effects of capsaicin on vascular cells from the perspectives of molecular mechanisms and signaling pathways, the overarching direction suggests its beneficial nature.

## 5. The Role of Capsaicin in Vascular Aging Related Disease

All cellular components depend on circulatory system in body, specifically the blood vessels, for the provision of oxygen and other vital nutrients transported in the blood. Similar to other organ systems, the vascular network undergoes aging, a phenomenon that inevitably leads to a gradual decline in its functional capabilities, resulting in various vascular diseases [2]. We have summarized the association of capsaicin in vascular aging related disease, aiming to investigate its role, especially in clinical trials or reports (Table 2).

**Table 2:** The clinical trials or reports of capsaicin in vascular aging related disease.

Disease	Clinical trials/reports
**Hypertension**	individuals with preference for spicy food exhibited lower blood pressure [124] no significant effect on blood pressure in meta-analysis of clinical trials [125]
**Coronary Artery Disease**	capsaicin lowers the risk of developing CAD [128] capsaicin reduces serum total cholesterol levels [129]
**Hypertrophic Cardiomyopathy**	capsaicin decreases cardiac hypertrophy and fibrosis [118]
**Cerebral Infarction**	capsaicin enhances the velocity of the middle cerebral artery, reduces the pulsatility index, improves collateral flow [148]
**Migraine**	capsaicin was used to establish migraine models [156,157] elevated levels of CGRP during migraine attacks [159]

## 5.1 Cardiovascular System

### 5.1.1 Hypertension

Hypertension, a pivotal factor contributing significantly to the rising prevalence of cerebrovascular accidents, ischemic heart disease, and renal dysfunction [120]. Lowering high blood pressure decreases the risk of developing cardiovascular events. Epidemiological investigations have demonstrated a relevance between consumption of chili pepper and normal blood pressure levels [121, 122]. However, the effects of capsaicin on hypertension are not universally acknowledged or consistently agreed upon. Animal studies indicate the activation of TRPV1 by dietary capsaicin facilitates endothelium-mediated vasorelaxation in WT mice. Prolonged TRPV1 activation not only enhances vasorelaxation but also effectively lowers blood pressure levels in genetically predisposed hypertensive rats [36]. Moreover, pretreatment of the paraventricular nucleus with capsaicin can mitigate blood pressure increases and reduce heart rate elevation in Dahl salt-sensitive hypertensive rats subjected to a high-salt diet [123]. In clinical trials, individuals with preference for spicy food exhibited lower salt intake and blood pressure compared to those who disliked spicy food, suggesting that enjoyment of spicy foods may enhance salty taste and thus reduce salt consumption [124]. Nevertheless, a meta-analysis of clinical trials indicates that capsaicin had no significant effect on blood pressure [125]. Besides, study shows TRPV1 activation can decrease the glomerular filtration rate, and subsequently elevate arterial blood pressure [126]. Given the confounding factors across various studies, more rigorous research is needed to dig out the efficacy and safety profile of capsaicin supplementation in regulating blood pressure. But most studies still correlate capsaicin with lowering high blood pressure.

### 5.1.2 Coronary Artery Disease

Atherosclerosis is the primary underlying cause of coronary artery disease (CAD), myocardial infarction, cerebrovascular accidents, and renal pathologies, the characteristics of which are chronic inflammation and progressive deposition of plaques [127]. Investigations demonstrate that capsaicin can reduce plaque area [60], thereby lowering the risk of developing CAD [128]. Additionally, a meta-analysis shows that dietary capsaicin supplementation reduces serum total cholesterol levels, which is an important promoting factor of atherosclerosis [129]. Delving into the underlying mechanisms, prolonged TRPV1 activation significantly diminishes lipid accumulation and atherosclerotic plaques in ApoE^-/-^ mice fed a high-fat diet, whereas no such effect is observed in ApoE^-/-^ and TRPV1^-/-^ mice under the same dietary conditions [34]. On the other side, the activation of TRPV1 can enhance the PKA and UCP2 expression, mitigates coronary dysfunction by alleviating endothelial mitochondrial dysfunction [114]. Additionally, capsaicin’s activation of TRPV1 can induce autophagy, hindes foam cell formation in VSMCs [106]. And the formation of foam cells is a crucial initial stage in atherosclerosis [130]. These underscore the potential role of TRPV1 in mitigating high-fat diet-induced atherosclerosis. Besides, it’s identified that capsaicin also can ameliorate atherosclerosis through modulation of gut microbiota composition [131, 132]. Given the substantial evidence indicating that capsaicin-mediated TRPV1 activation can diminish lipid accumulation and attenuate atherosclerotic lesion formation, researchers have devised a photothermal switch for TRPV1 signaling, which can be opened to decrease lipid storage and plaque formation in mice [133].

Importantly, disruption of plaques in atherosclerosis may lead to critical stenoses, causing many acute coronary syndromes (ACS) [134]. The vasodilatory effects of CGRP on coronary arteries have been frequently reported. Stimulating TRPV1 to release CGRP within coronary arteries contributes to mitigating the risk of CAD, notably by enhancing coronary flow and promoting arterial dilation [135]. Furthermore, studies conclude that capsaicin-sensitive CGRP contributes approximately 30% to coronary flow compared with the control group [136]. TRPV1 also impacts the coupling of myocardial blood flow to cardiac metabolism through NO and BK channel-dependent pathway [137]. Hypoxic-ischemic conditions frequently result in the apoptosis and necrosis of cardiomyocytes. Notably, the deletion of TRPV1 gene adversely affects cardiac recovery function following ischemia/reperfusion injury [138]. However, dietary capsaicin may protect myocardial infarction by inhibiting cardiomyocyte ferroptosis through activating myocardial TRPV1 [139]. Beyond myocardial damage from acute ischemia and hypoxia, reperfusion of ischemic tissue can also lead to myocardial injury within the reperfusion zone. Capsaicin can activate TRPV1 localized in cardiomyocyte mitochondria, regulating mitochondrial membrane potential to limit reperfusion injury [140]. Some studies show, capsaicin alone fails to elicit contractions in coronary artery, but prior treatment with proinflammatory prostaglandin-thromboxane agonists possibly lead to coronary vasospasm, which can exacerbate myocardial infarction and hinder post-myocardial infarction recovery [141]. The founding unmasks capsaicin's vasoconstrictive potential. However, the majority of research still favors the notion that capsaicin is beneficial in the prevention and treatment of myocardial infarction.

### 5.1.3 Hypertrophic Cardiomyopathy

Elevated mechanical stress on the heart, exemplified by hypertension, induces inflammatory response, ultimately leading to hypertrophic cardiomyopathy [142, 143]. Studies have shown that chronic consumption of capsaicin decreases cardiac hypertrophy and fibrosis induced by long-term high-salt diets [118], chronic infusion of capsaicin significantly improves cardiac hypertrophy and heart rate in rats [24]. One mechanism proposed is the activation of TRPV1, which ameliorates the deleterious effects on the efficiency of Complex I oxidative phosphorylation [144]. Another study indicates, in the condition of pressure overload, capsaicin can attenuate the upregulation of TGF-β, CTGF, and the phosphorylation of Smad2/3, thereby combat cardiac hypertrophy and fibrosis [145]. Conversely, some studies suggest that TRPV1 activation may induce cardiac hypertrophy through the upregulation of MAPK signaling pathway and increased intracellular polyamine levels [146, 147]. Overall, recent findings continue to support the protective effects of capsaicin against cardiac hypertrophy.

## 5.2 Cerebrovascular System

### 5.2.1 Cerebral Infarction and Vascular Cognitive Impairment

Cerebral infarction arises when there is impaired cerebral blood circulation, representing a significant cause of mortality. Clinical trials demonstrate that oral capsaicin enhances the velocity of blood flow in middle cerebral artery while reducing pulsatility index, thereby improving collateral flow among healthy volunteers [148]. These results have been replicated in patient samples with risk factors for cerebral infarction [149], suggesting a promising potential for capsaicin in rehabilitative treatment following acute ischemic strokes. One sequela of cerebral infarction is vascular cognitive impairment (VCI), which is another prevalent cause of dementia after Alzheimer's disease. VCI includes various cognitive disorders that are related to cerebrovascular injury, spanning from mild cognitive decline to severe dementia [150]. The roles of TRPV1 channels and capsaicin in VCI have garnered considerable attention. Direct administration of capsaicin into the peri-infarct zone significantly reduces infarct volume and enhances neurological behavioral scores, as well as motor coordination function in a rat model [151]. Conversely, in chronic cerebral hypoperfusion model, rats treated with capsaicin showed significant alleviation of cognitive deficits and companied by notable improvement in disrupted endoplasmic reticulum-mitochondrial interactions [152]. Capsaicin’s neuroprotective effect depends on TRPV1 and is attributed to the downregulation of NMDA receptor expression and function [151]. However, the precise role and mechanisms remain unknown. One study has found that TRPV1 agonists, such as capsaicin, significantly diminish the neuroprotective effects of electroacupuncture pretreatment, which coincides with enhanced TRPV1 expression [153]. This suggests, TRPV1 antagonist, and absence of TRPV1 channels, may decrease detrimental inflammatory responses during ischemia-reperfusion injury, thus providing neuroprotection. Recent research posits that both TRPV1 agonists and antagonists can offer neuroprotection, contingent upon dosage and duration [154]. Therefore, further investigations are imperative to determine the optimal dose of capsaicin that maximizes its beneficial effects.

### 5.2.2 Migraine

Migraine, one of the most prevalent headache diseases, results from dysfunctions within the trigeminovascular system. In this review, we focus on vascular-related aspects of migraines, which are believed to arise from reactive vasodilation [155]. In clinical trials, capsaicin-induced dermal blood flow was used to simulate models of migraine, to assess target engagement [156, 157]. TRPV1 receptor activation induces CGRP release and enhances cerebral blood flow [158], both are thought to contribute to headache development. CGRP is recognized as a potent vasodilatory peptide, particularly active in cerebral circulation [47, 159]. The level of CGRP is elevated in external jugular venous blood during migraine attacks [160]. Female sex hormones, particularly 17β-estradiol, enhance vasodilation by increasing CGRP release, potentially explaining the higher prevalence of migraines among women [161]. In rats, capsaicin administration into the cisterna magna results in the upregulation of CGRP, triggering spontaneous pain behaviors. Conversely, the downregulation of capsaicin-induced CGRP dampens the activation of the trigeminovascular pathway [77]. Amounts of studies on the effect of CGRP and TRPV1 in migraine have made them as therapeutic targets for migraine treatment and prevention [162].

### 5.3 Renal Vascular System

The renal vasculature is critical for sustaining kidney function across distinct anatomical compartments, such as glomeruli, cortical peritubular capillaries, and vasa recta bundles, all participating in renal metabolism and blood pressure regulation [163]. Renovascular hypertension necessitates TRPV1 channel activation, which diminishes glomerular filtration rate, and ultimately increases arterial blood pressure [126].However, excessive salt intake triggers TRPV1 activation, which exerts a counter-regulatory function, mitigating salt-induced arterial pressure increases [164]. Furthermore, TRPV1 activation decreases renal perfusion pressure while increasing glomerular filtration rate and the excretion of sodium/water, thereby modulating renal hemodynamics and excretory functions [165].

On the other hand, the renal vascular system can delivere oxygen and nutrients. Injury to renal vessels can lead to acute ischemic renal injury and subsequent reperfusion injury. TRPV1 may have a protective impact on acute ischemic kidney injury. Researchers have constructed a rat model of acute I/R renal injury, wherein marked declines in renal function were observed. They found that treatment with capsaicin 30 minutes prior to ischemia attenuated I/R-induced renal dysfunction [166]. Moreover, TRPV1 activation can prevent renal damage and salt-induced hypertension following I/R injury through anti-inflammatory and antioxidant mechanisms [167].

### 5.4 Others

While much focus is placed on cardiovascular and cerebrovascular diseases involving major blood vessels, microvascular diseases that affect various tissues throughout the body also warrant attention. Research demonstrates that diabetic patients with microvascular dysfunction and no evidence of CAD experience similar annual cardiac mortality rates as those with known CAD [168]. This underscores the severe consequences of microvascular disease. Diabetic retinopathy exemplifies a severe microvascular complication of diabetes, primarily caused by hyperglycemia-induced oxidative stress and inflammation in retinal microvessels [169]. Capsaicin treatment can mitigate hyperpermeability of retinal microvessels and retinal neovascularization in diabetic retinopathy rat models [68]. Additionally, idiopathic pulmonary arterial hypertension (IPAH), with increased pulmonary vascular resistance [170], may also be influenced by capsaicin. Capsaicin can activate TRPV1 to increase cytosolic Ca2+ concentration, contributing to excessive proliferation of these cells in IPAH patients [171]. Furthermore, TRPV1 also identified in the SMCs of bladder arteriolar in postpubertal female mice, with a significant impact on regulating microcirculation within the female bladder and potentially participating in bladder inflammatory diseases [172].

## 6. The Role of other aspects of Pepper in Vascular Aging

Pepper contains a diverse array of compounds beyond capsaicin, which also significantly influences vascular aging. Research indicates that capsiate inhibits proliferation, chemotactic migration, and capillary-like network formation of primary human ECs induced by VEGF. This suggests that capsiate may serve as an effective means to hinder pathological angiogenesis and associated vascular permeability [57]. The role of dihydrocapsaicin has garnered increasing attention. Studies show that dihydrocapsaicin treatment significantly improves neuronal density, decreases infarct volume, mediates angiogenesis, and facilitates functional recovery after ischemic stroke [173, 174], by attenuating oxidative stress and inflammation [175]. Furthermore, dihydrocapsaicin suppresses interferon gene stimulant-mediated accumulation of ROS and the NLRP3 inflammasome, alleviating apoptosis following ischemia-reperfusion injury [176].

## 7. The clinical use of capsaicin and TRPV1 agonist

Capsaicin’s function has been the subject of research for over a century, yet the development of capsaicin-based applications remains limited. The initial sensation that people notice is its spiciness and pain. Uniquely, TRPV1 once activated by capsaicin, comes to a prolonged refractory state, making previously excited neurons less responsive to a spectrum of stimuli, encompassing mechanical pressure, endogenous and exogenous pain, as well as proinflammatory agents [18]. As a result, capsaicin is frequently used as a local analgesic. The US Food and Drug Administration has granted approval of a capsaicin dermal analgesic patch, which is also authorized in the EU to manage peripheral neuropathic pain in adults, either as a monotherapy or in conjunction with other therapeutic agents [177]. The patch has demonstrated both safety and efficacy in managing neuropathic pain [178]. Furthermore, capsaicin exerts antioxidative, antitumor, antiulcer, anti-obesity, and anti-inflammatory effects, demonstrating potential as a treatment for cardiovascular, gastrointestinal, oncological disorders, and obesity. However, ongoing research is focused on determining its applicability in various clinical conditions.

TRPV1, a polymodal protein intricately tied to the genesis of pain, is receiving increasing attention. Both pharmacological and genetic investigations have solidified its role as a pivotal therapeutic target in various preclinical models of chronic pain, such as cancer-related pain, neuropathic pain, postoperative pain, and musculoskeletal pain [179]. TRPV1-directed medications could soon emerge as the next frontier in analgesics, offering relief for a diverse array of painful conditions. Moreover, TRPV1 has shown robust pathogenetic correlation with neurodegenerative diseases, particularly Alzheimer's disease [180], Parkinson's disease [181] and multiple sclerosis [182], through the regulation of neuroinflammation. TRPV1 agonists are also increasingly being a promising avenue in managing chronic conditions, particularly neurodegenerative diseases [183]. What’s more, TRPV1 has been implicated in the regulation of weight, pancreatic function, hormone secretion and thermogenesis, which suggests the potential therapeutic implications of targeting this channel [184].

In summary, the clinical development of capsaicin and TRPV1 agonists have achieved notable successes and has found widespread application in the treatment of pain. Additionally, they have demonstrated unique efficacy in treating other systemic diseases, such as metabolic disorders, neurodegenerative diseases, and gastrointestinal diseases. In the future, we believe their development is poised to increase.

## 8. Conclusion

Pepper, a natural plant and a common culinary ingredient worldwide, has garnered attention for its potential health benefits. As research on peppers deepens, the effects of capsaicin—the primary bioactive component—on human health are increasingly elucidated. Capsaicin is known to enhance metabolism, combat inflammation, and provide analgesia. Given that vascular aging is associated with chronic inflammation and metabolic disorders, capsaicin may hold unique potential in mitigating vascular aging. This review summarizes the role of capsaicin in vascular aging, beginning with molecular and cellular mechanisms, and extending to phenotypic changes. Evidence suggests that capsaicin and the activation of TRPV1 can inhibit the molecular pathways and signaling channels responsible for vascular cellular aging, thereby reducing migration, proliferation, and inflammation of senescent cells, and mitigating vascular aging phenotypes such as endothelial dysfunction and atherosclerosis. About vascular aging-related diseases, such as hypertension, coronary heart disease, and cerebral infarction, numerous studies indicate that capsaicin may delay disease progression. However, a minority of studies have reported adverse effects, such as coronary vasospasm leading to ischemia, which may be attributed to dosage and timing of pretreatment. Notably, while capsaicin and TRPV1 activation have been identified as potential triggers for migraines, they are also being explored as therapeutic targets for migraine treatment. Considering all discussed aspects, we believe in the substantial potential of capsaicin to combat vascular aging, warranting further trials to identify the beneficial effects of dietary pepper consumption or capsaicin formulations. Currently, there are limited effective treatments for vascular aging, and we anticipate that capsaicin and TRPV1 agonists may emerge as promising dietary strategies and efficacious drugs in the field of vascular aging.
